# Stereotyping and the treatment of missing data for drug and alcohol clinical trials

**DOI:** 10.1186/1747-597X-4-2

**Published:** 2009-02-18

**Authors:** Stephan Arndt

**Affiliations:** 1 Department of Psychiatry, Carver College of Medicine, University of Iowa, Iowa City, IA 52242, USA; 2 Department of Biostatistics, College of Public Health, University of Iowa, Iowa City, IA 52242, USA; 3 Iowa Consortium for Substance Abuse Research and Evaluation, Iowa City, IA 52242, USA

## Abstract

Stigma and stereotyping of marginalized groups often is insidious and shows up in unlikely places, for instance in how clinical trials consider dropouts in treatment research. A surprising number of studies presume that people who do not complete the study protocol relapse and code their data as if they had been observed. There is no good statistical rationale for this treatment of missing data and numerous and more defensible alternative methods are available. We need to be mindful about our attitudes and preconceptions about the people we are intending to help. There is no good reason to continue to support science built on this scientifically indefensible stereotyping, however unintentional.

## Background

Stigma and stereotyping of marginalized groups is alive and well in the 21^st ^century. Oftentimes, it is insidious. As researchers, we sometimes like to think that negative attitudes and bias are the afflictions of the less enlightened. Like addiction though, these afflictions sometimes show up in the least likely places.

## Clinical trials and missing data

A fair number of clients drop out of treatment and clinical trial studies. We typically do not know why subjects drop out; whether or not they are using again and if they are, at what level; and can make no direct assessments about outcomes. Their data are missing. How clinical trials in addiction research treat missing data depends on presumptions about why the data are missing. Too often, we pass over the assumptions because they are imbedded in the analysis. Even when the presumptions are explicit, it is sometimes too easy to skim over the Methods Sections of papers and not question the underlying assumptions of the statistical analyses.

A close examination of the assumptions in a surprisingly large number of published studies finds what can only be described as stigma and stereotyping of individuals because they are no longer participating in studies. There are several options for how the dropouts and their data are handled. The decision depends on the intent of the study and presumptions about why the data are missing.

To find out how research reports in substance abuse/dependence clinical trials generally dealt with their missing data, I did an informal review of recent clinical trials. I selected 4 prominent journals (i.e. *Alcoholism: Clinical and Experimental Research, Alcohol and Alcoholism, Journal of Studies on Alcohol and *Drugs, *Journal of Substance Abuse Treatment*) dealing with substance misuse. For each journal, I went back from the most recent issues until I found 10 reports of clinical trials or went back 6 issues, whichever occurred first. This resulted in 34 articles on clinical trials. I reviewed the reports' method of handling the missing data. Of the 34 reports, 28 provided enough information to tell what they did and 6 did not. A few papers analyzed their data more than one way.

Missing data causes a problem for some analysis methods that require complete data. One solution is to fill in or impute the missing data and just go ahead with the full sample pretending nothing was missing. The full sample is called the "intent-to-treat sample" and this method of filling in the missing data is called a "static imputation." Static imputation introduces very restrictive assumptions about the missing data, as well as several potential sources of bias in the results. However unintentional, the numbers inserted in the data to fill the missing information are based on the researchers' assumptions about the research participant as if he or she would have been assessed. More bluntly, the imputed data are the researchers' guesses. Nearly a third (n = 11; 32.4%) of the recent 34 clinical trial reports I read used static imputation, filling in the missing data with a "best guess." All but two assumed that the client had relapsed or returned to baseline levels of use. The remaining two used the last observation to fill in the missing data on subsequent assessments.

There is little statistical reason for using any static imputation method [1-3] so what might drive the decision to assume that all dropouts are relapsing? There may be a few good reasons. One might be to make the current analysis replicate an old study done before more modern methods of dealing with missing data were available. Another reason, and sometimes given, is that filling in the missing outcomes as "relapsed" makes the analysis more "conservative". That reasoning assumes that there are more dropouts in the treatment arm than in the comparison arm. However, a researcher would not know that before hand and the method for treating missing data should be specified a priori [4]. Furthermore, this method is not more conservative from a statistical perspective since it does not add in any error associated with not knowing what the real outcomes were. The sample size of real observations is also artificially inflated.

Since the mid 1980s, there have been considerable advancements in the statistical analysis of data with missing values. For example, the technique of multiple imputation introduced in the mid 1980s [5,6]. This method explicitly adjusts the error terms for the uncertainty surrounding the missing data. Other likelihood methods are available [7]. Still other options exist, for example simply using a statistical method that does not require complete data on all subjects – random regression, mixed models, or generalized estimating equations, survival analysis. These methods have been available since at least the late 1980s. Only 7 of the 34 articles (20.6%) indicated one of these more appropriate statistical treatments.

## Conclusion

Many published papers explicitly assume with no supporting information that clients initiating treatment but not completing the study protocol, relapse. Aside from the bias that this might introduce into the science, this practice supports, without basis, a negative message. We need to be mindful about our attitudes and preconceptions about the people we are intending to help. There is no good reason to continue to support science built on this unintentional stereotyping.

## Competing interests

The author declares that they have no competing interests.

## References

[B1] Fielding S, Maclennan G, Cook J, Ramsay C (2008). A review of RCTs in four medical journals to assess the use of imputation to overcome missing data in quality of life outcomes. Trials.

[B2] Figueredo AJ, Mcknight PE, Mcknight KM, Sidani S (2000). Multivariate modeling of missing data within and across assessment waves. Addiction.

[B3] Little R, Yau L (1996). Intent-to-treat analysis for longitudinal studies with drop-outs. Biometrics.

[B4] Hedden S, Woolson R, Malcolm R (2008). A comparison of missing data methods for hypothesis tests of the treatment effect in substance abuse clinical trials: a Monte-Carlo simulation study. Subst Abuse Treat Prev Policy.

[B5] Little RJA, Rubin DB (1987). Statistical Analysis with Missing Data.

[B6] Rubin DR (1987). Multiple Imputation for Nonresponse in Surveys.

[B7] Schafer JL (1997). Analysis of Incomplete Multivariate Data.

